# Burden of disease and adaptation to life in patients with Crohn’s perianal fistula: a qualitative exploration

**DOI:** 10.1186/s12955-020-01622-7

**Published:** 2020-11-20

**Authors:** Samuel O. Adegbola, Lesley Dibley, Kapil Sahnan, Tiffany Wade, Azmina Verjee, Rachel Sawyer, Sameer Mannick, Damian McCluskey, Nuha Yassin, Robin K. S. Phillips, Philip J. Tozer, Christine Norton, Ailsa L. Hart

**Affiliations:** 1 Robin Phillips Fistula Research Unit, St Mark’s Hospital and Academic Institute, Watford Road, Harrow, HA1 3UJ Middlesex UK; 2 Department of Surgery and Cancer, Imperial College London, St Mary’s Hospital, Praed Street, London, W2 1NY UK; 3 Faculty of Education, Health and Human Sciences, University of Greenwich, London, UK; 4 CAF-QoL Patient Representative, London, UK; 5 Florence Nightingale Faculty of Nursing, Midwifery and Palliative Care, King’s College, London, UK

**Keywords:** Crohn’s anal fistula, Patient reported outcomes, Quality of life

## Abstract

**Background:**

Perianal fistulas are a challenging manifestation of Crohn’s disease. Best medical and surgical therapy results in only about a third of patients remaining in remission at one year on maintenance treatment and sustained healing is often elusive. There is little published data on patient perspective of living with the condition or coping strategies in the face of non-curative/non-definitive treatment. We aimed to understand the experience of living with perianal fistula(s) and their impact on quality of life and routine functioning.

**Methods:**

This exploratory qualitative study used purposive sampling to recruit participants with current / previous diagnosis of Crohn’s anal fistulas, from national IBD / bowel disease charities. The “standards for reporting qualitative research” (SRQR) recommendations were followed. Unstructured individual face-to-face interviews were audio recorded, transcribed and analysed thematically. Early themes were reviewed by the study team including patient advocates, clinicians and qualitative researchers.

**Results:**

Twelve interviews were conducted, achieving apparent data saturation. Three broad themes were uncovered: *Burden of symptoms*; *Burden of treatment*; and *Impact on emotional, physical and social well-being*. Each included several sub-themes, with considerable interplay between these. The impact of perianal fistula(s) on patients with CD is intense and wide reaching, negatively affecting intimate, close and social relationships. Fistulas cause losses in life and work-related opportunities, and treatments can be difficult to tolerate.

**Conclusion:**

Crohn’s perianal fistulas exert a heavy negative physical and emotional impact on patients. These findings will inform development of a patient reported outcome measure to assess treatment effectiveness and quality of life for patients living with this challenging condition.

I think it just affects everything. It affects what I wear, it affects what I do, it affects my marriage, it affects everything (P2).

## Introduction

Perianal Crohn’s fistula(s) affect a third of patients with Crohn’s disease (CD). Fistulas often represent an aggressive phenotype of CD [1, 2], and follow a chronic course with symptoms including anal pain and purulent discharge. Despite best medical treatment a significant number of patients either never achieve response or subsequently lose response to biologic treatments. Only a third of patients with Crohn’s perianal fistulas which close on induction, remain in remission at one year on maintenance treatment, and this number falls further with time [3]. Surgical options include drainage and seton insertion (which minimises sepsis) and definitive options (e.g. fistula plug/glues, advancement flap, LIFT–ligation of intersphincteric tract and more novel sphincter-sparing techniques). These, however, tend to fare little better than medical therapy alone and whilst combined surgical and medical therapy offers improved benefits, cure often remains elusive. Most patients experience recurrence or persistence and a relapsing–remitting course of fistula activity, which is likely to severely impair quality of life.

The only current validated clinical assessment tool designed to assess Crohn’s perianal fistula activity is the Perianal Disease Activity Index (PDAI) [4]. It assesses pain, restriction of activities, restriction of sexual activities and perianal disease severity (discharge, disease type and induration). Items are scored on a 0 (no problem) to 4 (severe problem) Likert scale [4]. A core weakness of the PDAI is the lack of patient involvement during development, and hence it only reflects what clinicians view as important. Consequently, it does not assess the global quality of life impact on patients, and its relevance to what patients consider to represent successful treatment of fistulas is unknown. There is little evidence available on patients’ experiences of perianal fistulas in CD, but one early exploratory study interviewed patients with either CD or idiopathic perianal fistulas, and highlighted the extensive impact of these fistulas beyond pain and restriction of sexual activity [5]. The purpose of this investigation was to obtain detailed information from patients with Crohn’s disease, describing the experience of living with perianal fistula(s) and the impact it has on their quality of life and routine functioning.

## Materials and methods

As part of a larger project to develop a new Crohn’s fistula patient-reported outcome measure (PROM), we conducted an exploratory qualitative study to understand the experiences of those living with CD-related perianal fistulas [6–9]. Established qualitative techniques [8] such as purposive sampling, small sample sizes, narrative methods of data collection, and thematic analysis of data were employed. The “standards for reporting qualitative research” (SRQR) recommendations were followed [10].

The research question was: What are Crohn’s disease patients’ experiences of living with perianal fistula?

### Recruitment/sampling

Using purposive sampling, community-dwelling individuals with experience of CD-related perianal fistulas were recruited from the membership of collaborating specialist charities in the United Kingdom (Crohn’s & Colitis UK; ForCrohns; Bowel Disease Research Foundation). Advertisements were released online and via social media by these charities.

### Inclusion/exclusion criteria

Inclusion criteria were: aged > 16, living in the UK, self-reported diagnosis of Crohn’s disease with current experience of perianal fistula; ability to speak, understand, read and write in English language; ability to give informed consent. Patients without a diagnosis of Crohn’s disease were excluded.

### Data collection methods

Individual unstructured face to face, video-calling or telephone interviews were conducted, according to participant preference. Following introductory procedures (consent, collecting demographic and disease classification details), the interviewer prompted each participant to ‘*Tell me what life is like for you with a perianal fistula*.’ Follow-up questions and prompts were guided by the unfolding dialogue, thus enabling the participant to freely address their own fistula-related concerns. The aim of the interview was to secure an in-depth understanding of the experience of living with Crohn’s perianal fistula, including the impact of fistula diagnosis and subsequent surgical and / or medical treatments. Interviews were recorded on a digital audio device and transcribed and anonymised by an independent professional.

### Data analysis

All anonymised transcripts were returned to the study team (SA, LD, CN, PT, KS, TW, AH, NY and a patient/public involvement group – see below) for thematic analysis. Analysis was guided by the analytical hierarchy described by Spencer, Ritchie and O’Connor [11]; this involves several stages through which researchers individually identify themes and concepts within the data, then collaborate to agree themes, with analysis becoming increasingly interpretive as it progresses.

Each team member independently read the transcripts assigned to them, identified potential early themes and allocated provisional codes (labels). Each transcript was initially analysed by at least two team members. These early analyses were then submitted to a core group (LD, CN, SA, TW, PT) who subsequently discussed, refined codes, and agreed final themes.

## Ethical considerations

The study was approved by a university ethics committee (17/LO/1563). Informed consent was collected immediately prior to data collection. Interviews were conducted by two researchers, SA (a clinician) and LD (an experienced qualitative researcher).

### Patient and public involvement (PPI) team

Four members of Crohn’s and Colitis UK (CCUK), including an experienced PPI advocate were recruited to help with study design and data analysis. All had Crohn’s disease and previous or current experience of living with a perianal fistula. The PPI team helped with analysis and contributed to discussion, development and finalising of emerging themes.

## Results

**FIG. 1:**
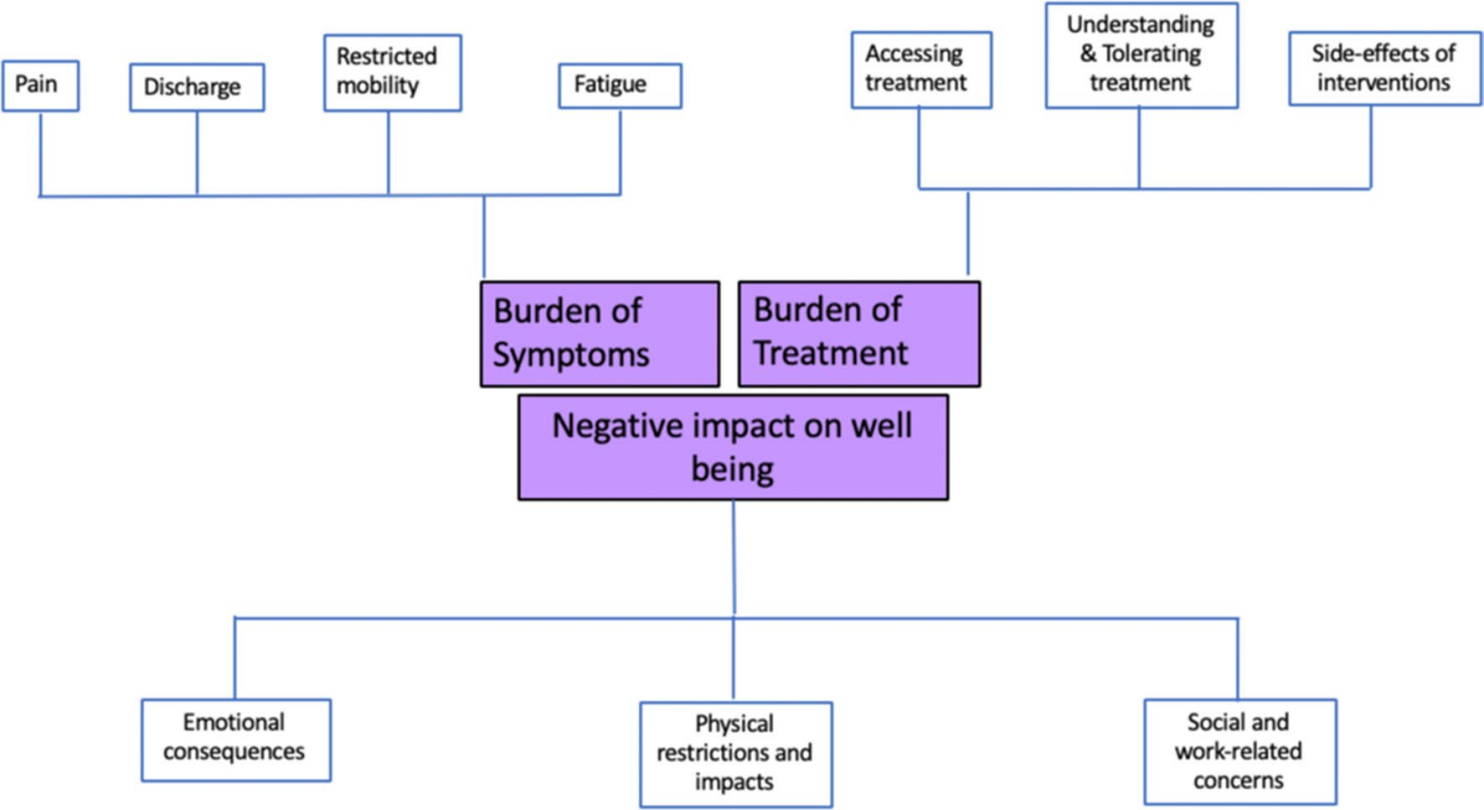
– Thematic depiction of the experiences of living with Crohn’s anal fistula: Themes are located in the central squares with the subthemes in the peripheral squares

Fourteen people with current or previous Crohn’s anal fistula participated (11 female, three male, aged 16–52). During interview, one (52-year-old, female) was found not to have a perianal fistula (but a previous abscess), and another (16-year-old female) had an entero-enteric fistula. Their data were not used in the analysis. Demographics for the 12 eligible participants included in the analysis are shown in Table 1. Participants provided very detailed and highly descriptive interviews; they frequently suggested that being able to divulge details about their life with Crohn’s anal fistula was very therapeutic. Following introduction of the trigger question, interviews were largely patient-led, with open questions and few interruptions from the interviewer. Median length of interview was 43mins (range 18–145 mins). Apparent data saturation was achieved after 10 interviews and confirmed in two subsequent interviews. During analysis, three themes, each with several sub-themes emerged from the transcripts, characterising the experience of living with Crohn’s anal fistula (Fig. 1):

- Burden of symptoms
- Burden of treatment
- Impact on emotional, physical and social well-being.

**Table 1:** Demographic details for participants taking part in the unstructured face to face interviews

Participant	Sex	Age	Duration of CD (years)	Duration of perianal fistula (years)	Previous fistula surgery (excluding abscess drainage)	Current Stoma	Medication for fistula
1	Male	26	13	4	Yes	Yes	Nil
2	Female	26	4	1	Yes	No	Antibiotics – cyclical
3	Female	34	14	5	Yes	Yes	Nil
4	Male	23	5	3	Yes	No	Anti-TNF
5	Male	23	2	2	Yes	No	Nil
6	Female	16	2	0.5	no	No	Nil
7	Female	39	5	5	Yes	No	Anti-TNF
8	Female	52	9	7	Yes	No	Anti-TNF
9	Female	37	6	2	Yes	No	AZT
10	Female	35	15	1	Yes	No	Anti-TNF + AZT
11	Female	43	19	14	Yes	No	Anti-TNF + AZT
12	Female	20	3	3	Yes	No	Anti-TNF + AZT

*AZT* Azathioprine, anti-TNF – biologic medication, specifically anti-TNF alpha drugs (infliximab / adalimumab), *nil* none

Verbatim excerpts from interviews illustrate each theme and sub-theme. Participants are represented by a Study ID number *(‘PXX’).*

### Theme 1: Burden of symptoms

This theme encompassed several key sub-themes, which were:

- Fistula-related pain
- Fistula-related discharge
- Restricted mobility due to the fistula
- Fistula-related fatigue

The predominant fistula-related symptoms experienced and expressed by every participant were pain and discharge, although the resulting perceived impact varied between participants.

#### (i) Fistula-related pain

Fistula-related pain was a prominent and significant source of impaired wellbeing, and participants described different pain sources. Pain was usually associated with a deterioration in health state, often coinciding with initial diagnosis of a fistula, the development of an abscess, or a flare up of fistula inflammation/infection. Pain was experienced as intense or sharp, and varied in frequency / occurrence with the number of local fistula complications (e.g. abscess, infection). Participants also described a more constant background discomfort associated with the mere presence of a fistula:

I know I haven’t had a day without pain for nearly four years… Pain contributes a lot to my life…’ (P7).

Overall, pain from the fistula had knock-on effects, which included needing powerful analgesia:

‘there’s no way that you could get past [the pain]. Because I couldn’t sit down or I was in like such bad pain that I was on Oramorph [oral morphine] and then you’ve probably got to be a special kind of, of intolerant or super tolerant person, I don’t know how you put it, to like to be able to function as normal when you’re on Oramorph’ (P1).

Pain further impacts on daily functioning, as discussed in subsection (ii), below.

#### (ii) Fistula-related discharge

All participants described the burden caused by fistula discharge. There were concerns about the quantity and smell of fistula discharge.

Quite frequently … probably once a day, or once every couple of days or whatever I have to sit on the toilet and release a load of discharge (P1).

Whether described as leakage, seepage or discharge, the output from fistulas caused embarrassment to participants, amid concerns about odour and visible leakage, effect on surrounding skin and clothing (discussed in Theme 3, subsection(i)). Persistent discharge associated with setons left some with the feeling of incontinence and increased the need for some degree of barrier protection (e.g. absorbent pads or gauze in their underwear) against skin irritation.

…there was this constant stuff coming out, and having to wear sanitary towels to try and make sure it didn’t leak through clothing … it was not an easy thing to try and manage (P7).

#### (iii) Restricted mobility due to fistula

Participants reported an overall impairment in mobility associated with fistula(s).

Factors contributing to this include the anatomical location, for instance—involving musculature and limiting range of mobility. Position-related pain or irritation around the fistulas was also brought on by sitting down/certain stances/walking/running:

On a day-to-day basis … I can’t really tolerate more than fifteen minutes sitting down (P11).

Similarly, irritation from setons in the perineum was exacerbated by certain sitting / standing positions or mobilising:

Because [the setons] are in my bum, it affects the muscles around my bum and it affects the way I can sit down (P4)

I don’t know whether it was the position of them, but I struggled to walk for much, for any length of time… (P7).

Scarring or inflammation in the tissues around the perineum were also reported to result in loss of ability to move in their premorbid manners and in some cases resulted in an antalgic gait. Discomfort caused by irritation from pads or gauze used to absorb leakage, played a role in restricting freedom of movement and patients often reported the need to constantly change positions as prolonged time in any particular position frequently resulted in discomfort.

#### (iv) Fistula-related fatigue

Participants often reported significant fatigue attributed to their constant experience of fistula pain. This pain-related fatigue was sometimes exacerbated by lethargy associated with use of analgesics. In these instances, patients reported being trapped in a cycle of pain—analgesia—fatigue—lethargy. Whilst a feeling of lethargy was attributed to underlying Crohn’s disease in some cases, it is important to note that some patients reported this being associated specifically with the anal fistula and not CD:

There’s like a lethargy which is resultant of the fistulas and also the Crohn’s, but I actually think that an awful lot of my lethargy came from the pain from the fistulas and also from, for example, not being able to get back to sleep because you are in pain because of the fistulas (P1).

There were reports of feeling exhausted due to the rigours of managing the mental and physical requirements of dealing with challenging fistulas. Some participants reported sleep disturbances due to waking up to change dressings or because of abrupt pain secondary to a seton knot catching within the fistula tract or on clothing material. Others described a general ‘wearing down’ due to the burden of symptoms and treatment:

You’re done in. Yes, it just wipes you mentally, which mentally makes you just want to lie in bed. There’s your physical side gone; you just don’t want to do anything (P12).

Furthermore, participants described the impact of fatigue on social and preferred activities, with their horizons becoming narrowed as fatigue related to fistula pain prevented them from pursuing hobbies and interests.

### Theme 2: Burden of disease treatment

Treatment of perianal Crohn’s fistulas is often multidisciplinary with long-term use of immunosuppressive or immunomodulating medications and surgical interventions for drainage or an attempt at definitive treatment. Often these therapies fail, and participants report undergoing a cycle of repeated burdensome treatments with elusive long-term cure. Under this theme, the key sub-themes were:

- Accessing treatment
- Understanding and tolerating treatment goals and processes
- Side effects of medical and surgical interventions

#### (i) Accessing treatment

Participants often described experiences of needing frequent access to treatment; multiple interactions with healthcare services were varied and included positive and negative perspectives. There was often a distinction between the experience of access to general and emergency services compared to specialist clinicians. When emergency care was required, participants often did not have access to tertiary care healthcare professionals (HCPs) with expert interest in Crohn’s anal fistula:

I have a problem sometimes if I go into hospital for an emergency operation, because the surgeon I [usually] see doesn’t work at the hospital with an A&E. He’s never there. (P4).

Participants reported some negative experiences when interacting with HCPs with poor understanding of the condition, its presentation, or acute exacerbations. Some participants reported receiving conflicting advice during management which led to loss of faith in the treating practitioner. Early identification of a developing or worsening abscess or fistula is essential to ensure timely, targeted intervention and to rescue and preserve deep tissues. However, participants reported that expert opinion was often lacking amongst receiving Accident & Emergency staff:

so, you go to A&E and what I tend to have found is that the majority of doctors won’t have necessarily seen [Crohn’s abscesses or fistulas] before and so, they don’t necessarily, they don’t necessarily know whether something is gearing up (P1).

In this space between the expert knowledge of the patient, and the non-specialist knowledge of the frontline staff, the patient’s condition can deteriorate. The challenges of self-presenting in emergency departments and difficulties accessing specialist opinions either at diagnosis, or at moments of exacerbation was a source of stress for participants, particularly if referral between hospital departments was either not expedited or there was a prolonged delay from time of referral from primary care:

My GP has told me many times, “There’s nothing that I can do to help you, you need to phone the hospital.” And then hospital appointments are so far, you know, few and far between…. (P3).

Delay in diagnosis, misdiagnosis, or lack of capacity to provide required specialist care could result in anxiety and loss of confidence in the healthcare service. Prompt access was often viewed positively, and positive established relationships with HCPs was linked to greater confidence in the care received.

#### (ii) Understanding and tolerating treatment goals and process

Participants reported that healthcare professionals often did not explain goals and methods of treatment coherently, or provide realistic expectations about setons and pain following surgery, or the extent of surgery:

It was a male doctor, a male colorectal surgeon that did it. And I didn’t realise that that operation was going to be more towards the vagina. And I had felt like I had just been butchered. That’s how I felt. It was so painful and I just remember thinking, “What have they done to me, what have they done to me?” And I never went back again. It was so painful (P3).

For this participant, the poor preparation and explanation of her surgery resulted in her withdrawing from health care support altogether. For others, mixed messages lead to confusion and difficulty knowing who to trust:

It’s just I have different teams telling me all different things. I have [one hospital] telling me ‘You need a [stoma] bag’. I have my surgeon telling me I need more setons in. I have my consultant at [another hospital] saying ‘You won't need a bag’. It’s just, I don’t have one opinion, I have four different and I don’t know who to trust. I don’t know who to say, “I’ll believe, I put my life in your hands and know that you’re going to make me better and do the best for me.” I just don’t feel like I have that with anyone (P12).

Consequently, participants often sought information from alternate sources, such as online searches and support groups to better understand the best treatment for them.

Others described more positive experiences, including developing rapport and trust with the surgeon who had performed several operations on them, and doctors following up when concerned about an individual’s wellbeing:

My doctor physically phoned me just to say, “Oh I was a bit concerned with you in clinic yesterday, how are you doing, is that alright, do we need to see you sooner than your next appointment that we booked?” (P5).

Understanding and having realistic expectations about interventions and outcomes was found to be important in helping patients cope with the possible relentlessness of CD-related fistulas. Several participants reported that the repeated treatments and the cycle of flare-ups gave them a sense of being on a constant treadmill of appointments, medication and surgical intervention. Some participants’ experiences revealed the perspective of fistula treatment being often onerous and seemingly futile. In particular, those with longstanding fistulas and multiple previous interventions seemed resigned to the lack of definitive cure and anticipated a future with repeated fistula relapses and remissions:

It makes me feel a bit hopeless, because I feel like there’s no hope of it getting fixed, ever (P9).

Participants also expressed frustration that HCPs themselves do not necessarily understand the impact of the very treatments they propose:

I am probably one hundred percent confident that no surgeon that has ever put a seton in, has had a seton themselves. (P1).

Failing to explain treatments adequately or showing lack of insight and empathy in respect of the impact and relentlessness of treatments, can be detrimental for some patients.

#### (iii) Side effects of medical and surgical interventions

Alongside dealing with the symptoms of and treatments for fistulas, participants revealed the burden of coping with the side effects of medical interventions. Concerns included systemic symptoms such as gastrointestinal disturbances, minor infections, rashes and headaches, and the potential risks of medication:

It has taken me fourteen years to accept medication. It has. There’s this thing in my mind where I think to myself, “Well, yes I know I’ve got the fistulas, but I cope. I work, I do this, I do that. Do I really want to take medication that’s going to increase my risk of cancer and it’s going to give me headaches and make me feel more fatigued? And, you know, I could suffer with anaphylactic shock.” And sometimes I see that the medicine will restrict my quality of life more. And I really worry about that, because nobody really knows until you take it. (P3).

The need for medications resulted in varying degrees of loss of independence, particularly for participants who were on infused medication requiring hospital visits for administrations.

The impact of surgery brought mixed responses from participants. Many viewed surgery as a positive necessity when, for example, they had developed an abscess requiring drainage. There were many strong negative sentiments about seton use, and some participants felt the presence of the seton was worse than having the fistula itself:

Every time that I’ve had one, the seton has caused a lot more discomfort than the abscess has given me in the first place…on two different occasions I’ve had such agony from the setons, I’ve had to cut the seton out myself’(P1).

My setons were placed a year ago. They’re the most uncomfortablest [sic] things I think I’ve ever experienced … they’re as thick as a straw and they’re knotted about eight times. You can imagine the thickness. The pain that they cause. They cause rubbing, they cause bleeding … they’ve caused little scars between my vagina and my bottom because they’re that thick, they’re literally … making you bleed throughout the day (P12).

The type of seton material used and method of securing it in place (degree of tightness and number of knots) varies between surgeons and can vary from silastic setons (bulkier) with multiple knots (or knotless), to suture material (finer in comparison) with smaller knots, with some newer setons having no knots at all. Participants reported a preference for suture material (which caused less discomfort), but the sporadic sensation of seton knots in the fistula caused pain, which often led to a degree of apprehension about mobility, sitting down or making sudden movements:

I don’t know whether it was the position of them, but I struggled to walk for any length of time’… (P7). ‘I used to have the bigger seton which I hated because it gave so much pain and now, I have the stitch seton which is better, but still uncomfortable because of all the knots… my latest seton, I think it’s too long (P9).

Participants also reported difficult experiences secondary to pain or discomfort when recovering from surgery, particularly following drainage or lay-open procedures. This post-operative period was often described as challenging particularly if there were large wounds after surgery requiring regular management:

Nobody really prepares you again for these operations. They tell you what it is and they didn’t really kind of explain every time, they didn’t explain the pain that I would feel (P3).

Furthermore, residual scarring and hardening of tissues around the buttock area from previous, often repeated, surgery also caused some negative experiences emotionally and physically:

I think the scarring around the fistulas creates this – not really a fear, but it just sets you in the mindset of if I push myself too far, it’s going to rip, something is going to happen. And you can feel that (P3).

Participants who were living with chronic fistulas present for 12 months or more, often expressed fear and uncertainty about possible future treatments. The formation of a temporary or permanent stoma, diverting faecal flow away from the fistula(s) is sometimes a necessary therapy when perianal disease becomes unmanageable. Proctectomy with removal of the rectum and anus may also be required. Some participants expressed an apprehension about needing a stoma in the future:

They have been talking about a stoma, so then you’re thinking, you know, is that something that I’ve got to consider… (P9).

Those who had undergone proctectomy and permanent stoma formation experienced this in the context of aggressive debilitating disease which had impacted negatively on quality of life. They had desired a more permanent resolution to their symptom burden due to the overwhelming negative experience of their fistula-related problems:

the fistulas got really bad to the point where I needed the stoma (P3).

Participants for whom the proctectomy proved curative for their fistula described positive outcomes, with relief being a common thread, as well as a sense of ‘having their life back’. They did however express that the decision-making was complicated as they perceived a lot of stigma related to the stoma.

### Theme 3: Negative Impact on Wellbeing

Several issues including the mere presence of a fistula and the symptoms related to it, and having to undergo fistula treatment, had various, often negative impacts on participant’s sense of wellbeing. The key sub-themes that emerged were:

- Emotional consequences
- Physical restrictions and impacts
- Social and work-related concerns

#### (i) Emotional consequences

Participants described wide-ranging emotional consequences of living with CD-related perianal fistulas, including impact on mental health, body image and self-confidence.

Mental health effects included worry, anxiety, feeling low or depressed, and mental exhaustion. Feelings of embarrassment often arose due to the fistula/seton and its location in an intimate area, leading to feelings of ‘not being normal’. Some participants described significant changes in personality and outlook and two participants described periods of significantly low mood and suicidal ideation driven by the burden of living with a fistula—both sought therapy and had or could overcome these periods:

The mental side of it, for me, is massive. It’s probably the hardest part for me to deal with. But I’m trying to deal with it every day. So that’s why it gets so hard. (P4).

Some participants felt more optimistic during periods of reduced symptom burden and decreased need for treatments, describing hopefulness, positive outlook, and acceptance. Positive interactions with health specialists, and strong support networks and relationships as well as online support groups often contributed to positive emotions:

There’s a fantastic closed group for fistula, American-based, but I think there’s about a thousand people in it, roughly and it’s amazing. There are people there who are just patients themselves but are very, very knowledgeable. And you will always get an answer for something or a suggestion or even if it’s just, “I understand.” (P11).

However, in some cases a necessitated increased reliance on others during periods of increased symptom burden led some to feelings of guilt and other negative emotions including feelings of depression.

As well as impacting on mental health, living with a fistula was also potentially detrimental to some individual’s self-confidence and body image. Participants described that the presence of the fistula in an intimate part of the body, with the often-associated (and sometimes faecal) discharge, resulted in feelings of altered body image:

[The fistula] does attack your self-confidence a little bit … I don’t really want to see it – I know what’s happened to me, I don’t want to see it, I don’t want to deal with it. Everyone else has got to dress me anyway, and do the dressings, and just tell me whether it’s good or bad. And I’ve kind of kept with that. And it does make me upset. I have seen it once, and I don’t like the appearance of it. (P2).

Scarring arising from surgical procedures also contributed to the sense of altered body image, while the presence of setons (particularly the bulkier silastic setons) contributed to participants feeling ‘abnormal’. These changes, combined with the need for pads/gauze or larger underwear for ‘hiding’ the changes, as well as restricted clothing options for some, led to feelings of reduced self-confidence and negative perceived body image:

You just want to look normal down there… I think because I have [the fistula] and it’s made me feel ugly, then it makes me start obsessing about other areas of my appearance (P9).

Many participants explained that they could not wear tight clothing, as this increased the discomfort they experienced from their fistula, and women in particular reported feeling limited in the choice of outfits available to them, further impacting on their sense of self, confidence and self-esteem:

You have to pick what clothing you wear – leggings can start infection, because they’re tight. Then, you’ve got to wear everything loose, which is a nightmare (P2).

There was considerable concern about risk of clothing being stained and the need to choose clothing colours specifically to mask any unpredictable discharge:

My knickers are constantly ruined because it’s, my, the leakage is, is going through my knickers or my brother’s boxers, if I wear his. Towels. I feel like towels just aren’t clean, because I’m constantly—it’s like yellow and green infection all over your belongings. I mean dark clothes are a definite. (P12).

Scarring, altered body image and low confidence could also have consequences for intimate relationships, with some participants volunteering emotional reasons explaining why they avoided sexual intercourse altogether:

I think you always have that anxiousness in your head, thinking, “Oh no, he’s going to, he’s just going to think I’m absolutely disgusting. (P12).

Participants reported high levels of anxiety around sexual intimacy, often related to the fear of leakage or unpleasant odour. This had a negative influence on emotional wellbeing, with restricted sexual freedom and physical pleasure often decreasing sexual desire.

The main problem is, for me, is the embarrassment if there was discharge. It’s happened before and it’s just like, it’s just put me to a point where I just say, “I’m so sorry,” and I just literally like just sat on the edge of the bed and just cried. (P4).

For many participants, from newly-weds through to those who had been married for many years, the lack of physical intimacy in their relationship was experienced as a huge loss.

#### (ii) Physical restrictions and impacts

In addition to the emotional impact, fistulas caused, they resulted in physical restrictions in activities of daily living and intimate activities, as well as impacting on cleanliness and hygiene. Restriction of simple daily functions like sitting down had knock on effects on wide-ranging activities of daily living. Walking and running were also sometimes affected in some participants leading to aversion and coping strategies, included limping, and avoiding walking long distances or running activities that would trigger discomfort, with negative impact on regular exercise and sporting activities, and certain activities (e.g. cycling, swimming) becoming impractical for some.

Well you learn to walk differently, you learn to sit differently, you learn to stand up differently. (P3).

I felt like I couldn’t [go and] swim. Whether I could or not, it didn’t feel hygienic to swim with the fistula because especially if it’s still leaking, it just doesn’t feel right (P7).

Driving was one thing that I really found difficult to begin with, just speed bumps, holes, you know, things like that (P5).

Fistulas were understood to negatively alter intimate sexual experiences, as participants described physical restrictions limiting freedom of movement and enjoyment, as well as pain from the fistula being exacerbated with intercourse:

We don’t do sex anymore…it’s like l am a born-again virgin, in a sense, that’s how I feel, because the pain, it just hurts (P11).

Setons also restricted freedom of movement during sexual activity:

With the seton again, you feel a bit embarrassed, especially if it’s a seton that hangs low or moves or anything. You know, you just can’t settle or enjoy (P3).

Participants often expressed a significant change in their daily physical routines to compensate for the physical restrictions, and also reported an increased level of ‘self-maintenance’ required because of the fistula. This was reflected in participants’ experiences of maintaining self-hygiene and cleanliness, leading to altered hygiene habits. Behavioural changes included frequent shower use and carrying spare underwear and/or a full change of clothing in case of accidents. This often led to prolonged time spent in the bathroom, increased toilet usage and in some cases almost ‘ritualistic’ toilet use brought on by having multiple fistulas:

I find a bit of a Catch 22 with it is, everyone tells you to keep it clean, keep it clean, keep it clean. But the area that it’s in and the fact that it’s draining pus…, that’s not an easy thing to do. (P5).

Increased toilet usage was sometimes described as necessary to avoid soiling accidents and public embarrassment. Some participants reported difficulty in maintaining a clean genital area, a struggle made worse by increased fistula discharge or multiple setons:

Trying to use toilet roll when you’ve got this mess of abscess and seton is just a nightmare, because it just all gets caught up’ (P7).

#### (iii) Social and work-related concerns

The presence of perianal fistula(s) was often described as adding a layer of complexity to managing and living with Crohn’s disease. The social and work-related concerns arising from having a fistula were revealed in participants’ descriptions of the effects on employment and finances, independence, social interactions, relationships, and development of coping strategies to deal with these.

Participants described having to take time off work/education usually due to fistula-related surgery, treatment or flare. Whilst participants struggled to quantify the losses, most spoke of a sense of loss of work options or potential, and concerns about job stability:

I’ve been off sick since February 2015. 'For the last few years prior to that, the holidays when you get four, five weeks’ holiday in a year, I was taking them in a sense as sick days, because I didn’t want to lose my job because I’m scared of who’s going to employ me with my sick record (P11).

Participants felt they had restricted options for the types of jobs they could manage, for example needing to avoid those involving prolonged sitting (or jobs involving driving). Some participants changed their occupation to avoid exacerbating fistula pain and discomfort, Similarly, younger patients reported problems with prolonged sitting in school:

It was just totally impossible for me to, to be able to sit for three hours to do an exam, so, I remember doing the exams while I was kneeling (P1).

There were also reports of workplace modifications, such as adjusted seating to suit a participant’s specific needs. For those participants who were working, many felt supported by their workplace:

I ended up missing quite a lot of work with the abscess and the fistula combined… I was so lucky, I had a very, very understanding boss, who was just brilliant … (P10).

Managing the fistula had financial implications, not least due to the increased use of pads, gauze, and underwear to maintain cleanliness:

It’s unbelievable. I’ve probably got through a box; how many boxes a week? Three, four boxes a week of pads. I’m constantly buying them (P12).

Participants also described loss of earnings related to the fistula due to the inability to work, or perceived reduction in career potential due to needing substantial time off work. Some participants also reported an increased dependency on friends and family for financial support as well as practical support which in some case led to a sense of loss of independence:

I haven’t had any salary since January. And so, I quite rely on family to get me by, and also, my partner’s income. It’s also affected, affecting even looking forwards around the type of job that I get, because I’m wary of getting a full-time job [in case I] fall back into this like boom and bust way of managing my healthcare (P1).

The uncertainty of fistula-related exacerbations had no respect for important periods of participants working / education lives, or indeed other personal milestones. Participants’ sense of ‘self’, arising from being independent, in control, and having choices in life, was negatively affected during periods of exacerbation and rescue treatments. The fistula was understood as robbing them of potential or ability to fulfil their dreams:

[The fistula] has a mind of its own and I’m along for the ride (P2).

Living with a fistula reduced social opportunity and some participants described avoidance of social situations due to a need for ‘toilet-mapping’, which felt restrictive. The presence of discharging fistula(s) raised the constant need for access to a suitable toilet, causing participants to always frequently look for facilities in new environments. This sometimes restricted options for going out, with participants preferring venues where the toilet facilities were easily accessible and clean.

So, I wouldn’t go there if there’s no toilet (P2).

I still went out with, with my daughter, but we often just used to go to cafés or whatever, where I knew there was toilets (P7).

Participants who experienced frequent exacerbation of fistula symptoms tended to reduce social interactions to avoid either the physical requirements (e.g. frequent toilet use, prolonged sitting) or the degree of pre-planning that would precede being able to attend. Others cancelled plans due to low mood or fatigue and decreased desire for social engagements, and in some cases, due to concern about stigmatising attitudes from others. Participants sometimes selectively limited disclosure of their illness as an attempt to avoid potential stigma:

I don’t really go out. Since having perianal disease, your social life as a twenty-year-old completely disappears. You can’t do anything because no one understands. (P12).

Participants reported varied experience of relationships secondary to having a fistula and these were both positive and negative. Some participants experienced increased closeness to family members or a close circle in whom they confided and who provided a support network subsequent to their diagnosis of Crohn’s anal fistula:

I genuinely do think that I probably am lucky to have a partner that is, not just understanding, has time to listen, but also probably understands it more than me.’ (P1)

A recurrent theme was apprehension about starting new relationships, particularly intimate ones, and these feelings often coincided with periods of fistula exacerbation or need for recurrent rescue treatments:

It affects your relationships with your friends. And family as well. Because it’s hidden as well, I find that I’m always pretending, you know, “I’m fine” is the two famous words. And just keep on smiling, even though if you feel really dire at times. Because people can’t see what’s going on inside, they just assume that everything is fine (P11).

To overcome these many negative fistula-related impacts, some participants developed coping strategies. These included seeking further information and understanding, as well as seeking support via family, or through groups and individuals with similar diagnosis, particularly online support groups:

Just to touch base with someone who’s been through a similar thing is always good for me to do (P5).

Career, work and personal aspirations were often modified due to symptoms and frequency of interventions. Discussion with work colleagues (particularly those in senior positions) sometimes resulted in positive modifications of work environments which helped participants. Prompt access to, and faith in, expert HCPs as well as successful interventions—including fine thread setons and medication which reduced fistula leakage—were described as positive experiences which enabled participants to cope and live better with the fistula:

I have had huge support from, particularly, my husband and from all the medical professions that I’ve been dealing with (P8).

In contrast, some participants described avoidance of coping mechanisms and negative emotions, and absence of a support network. Typically, these participants described some degree of withdrawal from society, with an inevitable negative impact on social interactions and development of new relationships. There were also reports of withdrawal and ‘distancing’ from established relationships with a pretence of coping well:

I basically try and make it so that no one thinks I’m having any issues, because to me, that’s the easiest way to deal with it, because then less people ask more questions. (P4).

## Discussion

This study reports the results of the first in-depth qualitative exploration into experiences of living with Crohn’s anal fistula. The rich data obtained provide a deeper understanding of the potential challenges facing patients. The experiences of patients with Crohn’s anal fistula are far-reaching and extend far beyond the fistula-related symptoms. Our results demonstrate that these experiences can be broadly divided into three categories: burden of symptoms, burden of treatment and impact on physical, emotional and social aspects of wellbeing. There is considerable interplay between these themes and the extent to which they were experienced varied from participant to participant (Fig. 1).

Providing accurate, in-depth and targeted information for patients at the point of fistula diagnosis is likely to be beneficial. Health care professionals could use Crohn’s anal fistula information leaflets, and visual representation of the patient’s fistula using novel imaging techniques which have been recently been described (e.g. 3D MRI and 3D printing of fistulas) [12, 13] to achieve this. Patients’ source of information and the psychological response to the diagnosis has a bearing on how they perceive their fistulas. Trusted healthcare professionals, support groups and online sources often help with building positive experience.

Following diagnosis, the nature of the symptoms and subsequent disease course or treatment that a patient receives, determines the experience that the patient subsequently has. Those with mild and less troublesome symptoms or who achieve early healing, can be anticipated to have a reasonably limited deterioration in their quality of life secondary to the fistulas. However, this study demonstrates that those with more chronic fistulas or significant symptoms, have a very different experience which is often restrictive and negative in nature. Areas affected can vary from restriction in daily activities, to decreased career and financial potential as well as significant restriction of factors that contribute to emotional, physical and social wellbeing. The unintended detrimental effects of setons may be reduced through thoughtful use of finer sized seton materials (with fewer knots) that are available to surgeons. Chronic illness adaptation requires successful negotiation to a new identity [14] and this can sometimes be challenging in Crohn’s disease due to unpredictable disease flares [15]. Referral to support groups (online / via registered charities) may help with the negotiation of this new identity. There is often a lack of systematic follow-up for these patients, with anecdotal inaccessibility to a durable and robust network of multidisciplinary healthcare professionals with the competency to guide and offer the medical, nursing and psychological support that may be required. Consensus guidelines on managing perianal Crohn’s fistulas now advocate a multidisciplinary approach as standard of care with some centres providing resources catered to delivering this [16].

Our findings demonstrate that the impact of Crohn’s disease anal fistulas on patients extends far beyond the limited clinical, physical and sexual activity focus of the PDAI [4]. There is no available tool which assesses clinical markers of treatment effectiveness as well as the global quality of life impact on patients, developed to current standards and with patient input. There is a need for a patient reported outcome measure (PROM) that encompasses the key aspects covering the range of patient experience as demonstrated in this study.

## Strengths and limitations

Individual, followed by team analysis and agreement, enhanced credibility of findings by ensuring data analysis and subsequent findings were not influenced by a lone researcher, but are a consensus of the study team. The study benefitted from robust input from a patient and public involvement group who assisted in reviewing anonymised transcripts and developing and refining early themes. A multidisciplinary steering group allowed for crucial stakeholder perspectives to be included in the analysis and review of emerging themes.

Limitations included inability to transfer findings to the elderly population of patients with CD fistulas, since the oldest study participant was 52 years old. A limitation of the study was the inability to transfer findings to the elderly population of patients with CD fistulas, since the oldest study participant was 52 years old.

## Conclusion

The impact of perianal fistula(s) on patients with CD is intense, leading to reductions in life opportunities and negatively affecting intimate, close and social relationships. Capturing patient experience that extends beyond disease-related symptoms improves understanding and gives insight into the overall impact of Crohn’s anal fistulas. Currently, no patient reported outcome measures are available for use in routine practice in Crohn’s fistula management and as a result, patient outcomes (other than disease-related symptoms of pain and discharge) are rarely captured [17]. The patient experience is invaluable in the development of a PROM for this condition which would benefit in monitoring and improving the care for individual patients [18–21]. Potential areas of use include routine practice, monitoring treatment efficacy, clinical trials, and telemedicine systems [6, 20, 22]. Work is currently underway to develop a Crohn’s Anal Fistula Quality of Life tool, using the information gathered in this study [23].
